# Respiratory Viral and Bacterial Factors That Influence Early Childhood Asthma

**DOI:** 10.3389/falgy.2021.692841

**Published:** 2021-07-22

**Authors:** Nontobeko Mthembu, Paul Ikwegbue, Frank Brombacher, Sabelo Hadebe

**Affiliations:** ^1^Division of Immunology, Department of Pathology, Faculty of Health Sciences, University of Cape Town, Cape Town, South Africa; ^2^Division of Immunology, Health Science Faculty, International Centre for Genetic Engineering and Biotechnology (ICGEB) and Institute of Infectious Diseases and Molecular Medicine (IDM), University of Cape Town, Cape Town, South Africa; ^3^Faculty of Health Sciences, Wellcome Centre for Infectious Diseases Research in Africa (CIDRI-Africa), Institute of Infectious Diseases and Molecular Medicine (IDM), University of Cape Town, Cape Town, South Africa

**Keywords:** T helper 2, bacteria, viruses, asthma, early development, lung function

## Abstract

Asthma is a chronic respiratory condition characterised by episodes of shortness of breath due to reduced airway flow. The disease is triggered by a hyperreactive immune response to innocuous allergens, leading to hyper inflammation, mucus production, changes in structural cells lining the airways, and airway hyperresponsiveness. Asthma, although present in adults, is considered as a childhood condition, with a total of about 6.2 million children aged 18 and below affected globally. There has been progress in understanding asthma heterogeneity in adults, which has led to better patient stratification and characterisation of multiple asthma endotypes with distinct, but overlapping inflammatory features. The asthma inflammatory profile in children is not well-defined and heterogeneity of the disease is less described. Although many factors such as genetics, food allergies, antibiotic usage, type of birth, and cigarette smoke exposure can influence asthma development particularly in children, respiratory infections are thought to be the major contributing factor in poor lung function and onset of the disease. In this review, we focus on viral and bacterial respiratory infections in the first 10 years of life that could influence development of asthma in children. We also review literature on inflammatory immune heterogeneity in asthmatic children and how this overlaps with early lung development, poor lung function and respiratory infections. Finally, we review animal studies that model early development of asthma and how these studies could inform future therapies and better understanding of this complex disease.

## Introduction

Respiratory infections are one of the leading causes of disease morbidity worldwide (1), with infectious agents being the major sources of lung disease mortality (2). Over the last 3 decades, respiratory infections have been reported as the greatest contributing factor to asthma exacerbation in both children and adults. The severity and frequency of exacerbations varies from mild to more serious bronchiolitis, wheezing, or pneumonia in the lower respiratory airway (3). What is puzzling in newborns who develop these symptoms is that the acquired maternal antibodies meant to protect at this early age, do not seem to function optimally leading to significant morbidity and mortality. In 2010, 5 million deaths were reported in children below the age of 5 years, of which 64% were due to infectious agents within 30 days of life (4).

Allergic asthma is a common and major childhood illness that is caused by a combination of several factors including genetics and environmental factors such as microbes, air pollutants, aeroallergens, food, and many other factors yet to be determined (5). This complex chronic lung disease is characterised by shortness of breath, coughing, bronchospasm, and wheezing sound, caused by pathophysiology of obstructed airway and hyperresponsiveness (6). The disease affects over 300 million people worldwide and in 2019 an estimate of 461,000 related deaths were reported according to WHO (7). In the USA, ~10% of school-aged children are affected by asthma with at least 95% of those children having developed the disease before the age of 5 years (8). Several infectious agents have been associated with increased burden of asthma and wheezing in newborns, including bacterial, fungal, and viral infections (9, 10). Viral respiratory infections are considered as the single greatest cause of mortality and economic burden (11). In the USA, conservative estimates of economic costs of viral infections was ~$25 billion per annum with the highest impact observed in young children (11). Apart from viruses, bacterial infections have also been associated with significant asthma burden, accounting for many wheezing illnesses that may lead to early childhood asthma (8).

Despite substantial progress made in the past two decades to better understand risk factors, environmental triggers, phenotypes, and pathophysiology of the disease, key disease determinants, exacerbation factors, and prevention strategies remain elusive. In genetically predisposed individuals, respiratory viral and bacterial infections have emerged as a major predictive factor in developing wheezing at an early age (12). The detection rates of these infectious agents are highest among infants with wheezing and recurrent illnesses compared to adult's counterpart with wheezing signs (9). Among viruses detected, human rhinovirus (RV), respiratory syncytial virus (RSV), enteroviruses, human bocavirus, influenza viruses, human parainfluenza viruses, and coronaviruses have all been associated with asthma exacerbations in humans (13). Rhinoviruses are a more common single trigger of acute asthma exacerbations in school-age children, accounting for up to 76% of wheezing episodes in this age group (9). On the other hand, RSV is recognised as a second most common respiratory virus connected to wheezing sound and subsequent development of asthma in the first few months of life (3, 14). Bacterial species that have been linked with asthma exacerbations include *Haemophilus influenza, Streptococcus pneumoniae, Moraxella catarrhalis, Mycoplasma pneumoniae, Chlamydia pneumoniae* (15).

Asthma is a disease that has always been characterised by the presence of T helper 2 (TH2) airway immune cells such as eosinophils and allergen-specific IgE production by plasma cells (16). More recent studies using in depth molecular techniques and immune profiling in the airways or bronchoalveolar lavage (BAL) fluid have shown a far more complex disease with distinct but overlapping endotypes (6, 17). These endotypes represent TH2 high, TH2 low, TH1 and TH17 types or mixed phenotype often associated with asthma severity and non-responsiveness to corticosteroids (discussed in later sections) (17, 18).

**Figure 1:**
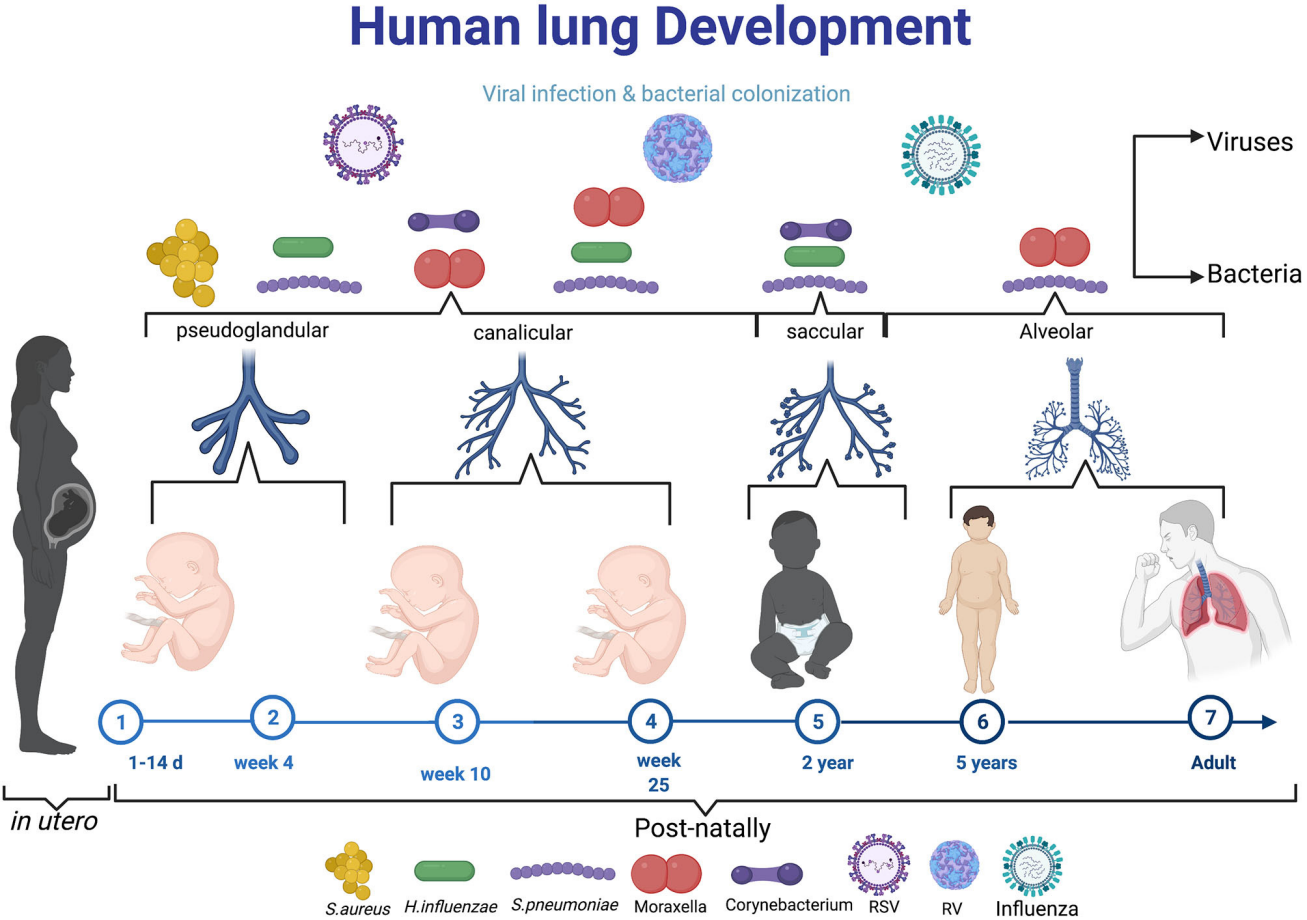
The diagram elaborating the neonatal exposure to viral and bacterial infections during the crucial stage of lung development. During pseudoglandular and canalicular of lung development (Day 1–25 week) where lung are thought to be more susceptible to early-life pathogens like Respiratory syncytial virus, *S. aureus*. From 25 week to 2 years (saccular), the most reported microbes in this age are RV, *H. influenza, S. pneumonae*, Moraxella, Corynebaterium, and then influenza virus can infect at time from birth to school age and adult. Created with BioRender.com.

During childhood (when the immune system is not fully mature), recurrent respiratory infections may induce impairment on the developing lung and alter its function through disruption of epithelial barrier integrity (Figure 1) (19). This could have long-term imprint in the lung and subsequent development of early childhood and late onset asthma (19). These epigenetic modifications and rapid hypomethylation of regulatory regions of TH2 genes at an early age suggests that early interventions during development in predisposed individuals may be beneficial therapeutically (20). Early immune education of the immature immune profile in the developing lung may serve as a critical window to prevent wheezing and asthma, however these studies are difficult to conduct in humans. Animal studies have shed light on how early respiratory infections can influence development of asthma at an early age. For instance, respiratory viral infected mice responding to aeroallergens sensitisation demonstrated increased and chronic airway responsiveness and inflammation (21). These observation suggests that viral induced infections in infancy may increase the risk and severity of asthma development at later stages of life (Figure 1). Whether this interaction is a direct effect of viral or/and bacterial replication in respiratory epithelial tissues or indirect effects emanating from certain secondary underlying conditions or factors that predispose individuals to atopy is still not clearly defined (22).

Clinical evidence from human studies and mouse studies supports a hypothesis that *utero* and postnatal inflammatory profile is TH2 biassed which may be the bases for development of asthma disease in children (23, 24). Respiratory viral and bacterial infection have been reported to promote early wheezing and later development of asthma with long term impacts on lung function. In this review, we focus on different respiratory viral and bacterial pathogens and their association with asthma exacerbation in children under the age of 10 years. We emphasise how inflammatory immune heterogeneity in asthmatic children overlaps with respiratory infections and poor lung function. We further highlight how animal studies could be a holy grail in uncovering early development of this heterogeneous and complex disease.

## Immunopathophysiology of Asthma

Asthma is characterised by shortness of breath and wheezing due to reduced air flow in terminal and smaller bronchial airways of the lung (25). The pathology of the disease is characterised by increased influx of granulocytes such as eosinophils, TH2 cells, increased circulating immunoglobulin E and mucus hypersecretion in the airways (26, 27). These symptoms are, in most cases, reversible upon bronchodilator or corticosteroid administration. However, over 5 decades ago Schwartz and colleagues first reported a phenomenon whereby this treatment was ineffective which they described as a “decrease in eosinopenic response to Cortisol and an accelerated plasma Cortisol clearance” (28). Since then, more studies have come out exploring steroid resistant asthma citing disease severity as a common feature among this sub population of asthmatics (29–31). A patient is considered to have steroid resistant asthma when the amount of air exhaled during forced expiratory volume per second (FEV_1_) cannot be rescued by 15% from baseline after steroid treatment over a period of 2 weeks (32, 33). Further investigations have also shown that these patients are characterised by a remarkably low eosinophilia and a prominent neutrophilic response (34, 35). Therefore, while asthmatics may present with similar symptoms, evidently the pathophysiology behind these similar clinical and physical presentation varies. The discovery of a non-eosinophilic type of asthma led to a change in paradigm in how asthma was being diagnosed and treated and resulted in further characterisation of the disease into multiple endotypes based on inflammatory granulocytes present and cytokines accompanying this inflammation (6, 35, 36).

## Immune Profile in Asthmatic Children

**Figure 2:**
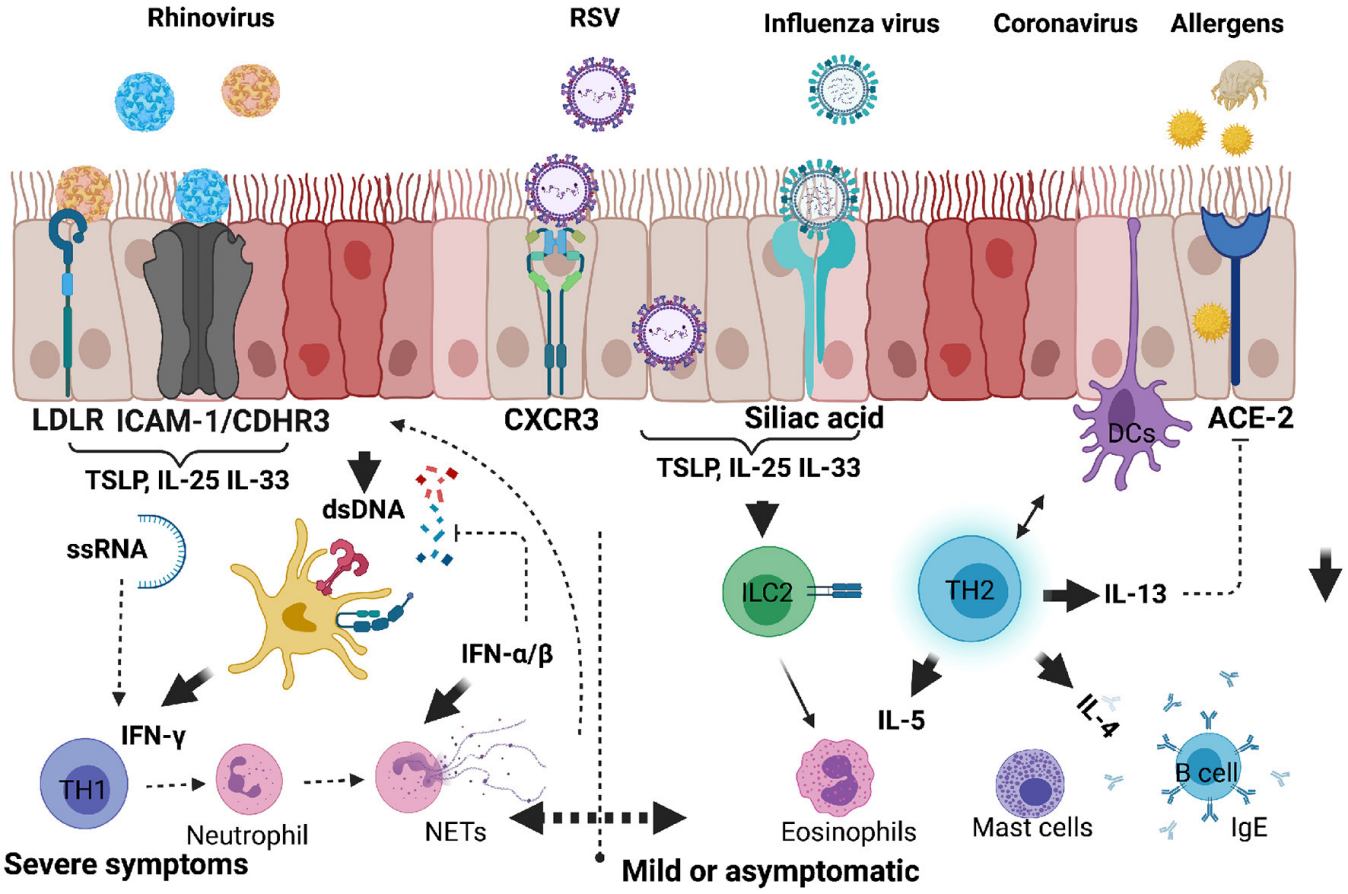
Respiratory viruses that infect upper and lower respiratory airways causing inflammatory response and exacerbating allergic asthma in children. When infectious agents such as viruses or allergens bind to their respective receptors on the surface of epithelial cells, they activate downstream signals. Upon these agents entering in asthma predisposed individuals, there is an increased tendency to produce proinflammatory cytokines such as IL-25, IL-33, or TSLP by epithelial cells. ILC2 are directly activated to produce IL-5 and IL-13, whereas TH2 cells are activated by DCs. Viral RNA or DNA can be detected by host nucleic acid receptors which help produce IFN-α/β and promote anti-viral TH1 cells producing IFN-γ. Dysregulated antiviral response can lead to neutrophil degranulation, NETosis and exacerbation of mild or moderate responses to severe form of allergic asthma. Created with BioRender.com.

**Figure 3:**
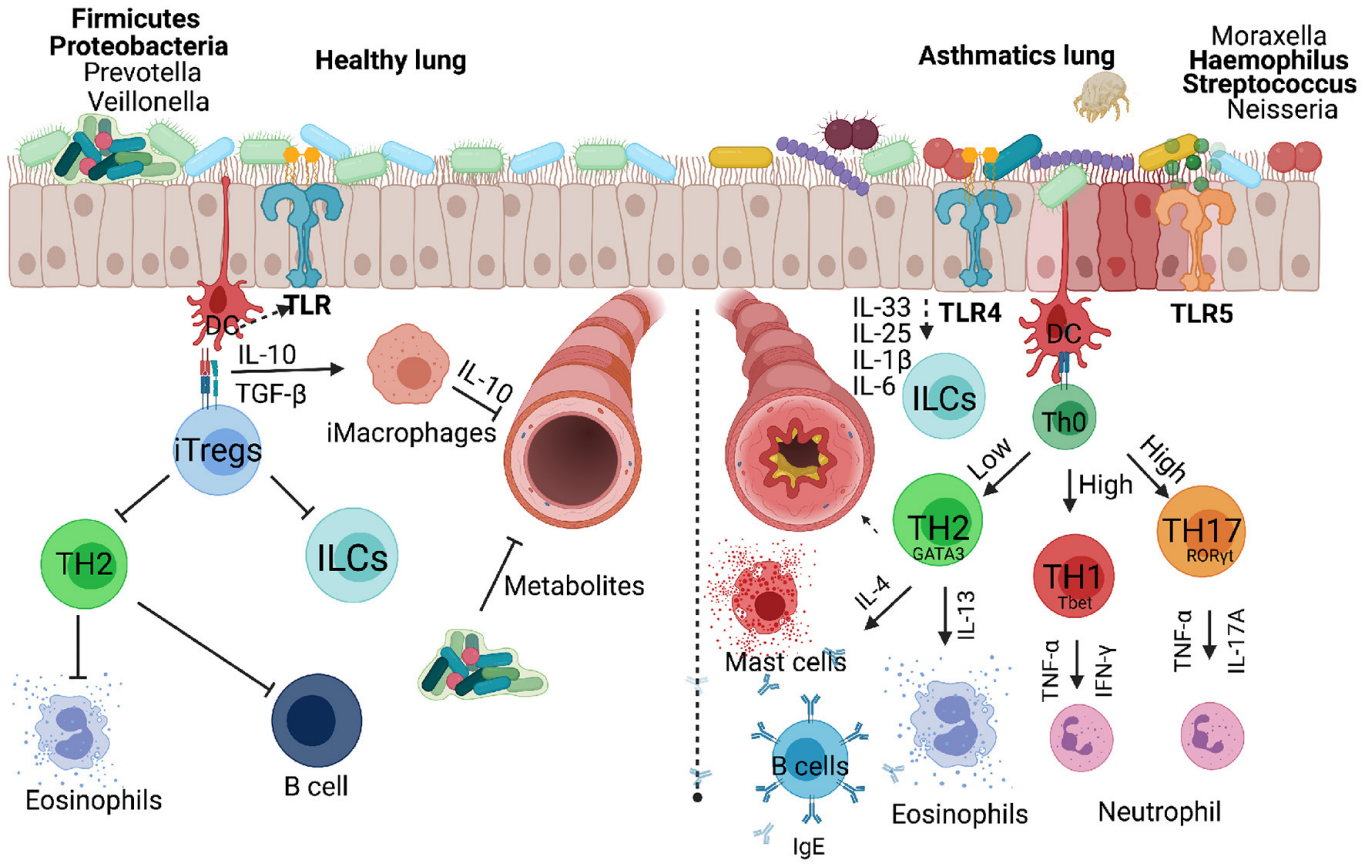
Respiratory bacteria and microbiota that infect or reside in the upper and lower respiratory airways either inhibiting or exacerbating development of allergic asthma in children. Microbiota dominated by Firmicutes and Proteobacteria protect a developing lung promoting secretion of metabolites that can inhibit infectious microbes. Inducible regulatory cells play a key role in countering TH2, innate lymphoid cells and B cells which contribute to asthma development. Invading microbes can either counter TH2 responses or exacerbate allergic asthma through activating TLRs signals that favour TH1 or TH17 responses associated with severe form of asthma. Created with BioRender.com.

Asthma is a heterogeneous disease common in both children and adults and present with multiple and variable inflammatory states. In more recent years there has been a renewed interest to characterise these heterogeneous immune inflammatory phenotypes in order to identify appropriate and personalised therapeutics for each disease phenotype. In adult asthma, the disease has largely been characterised as either mild/moderate or severe form (37). Mild and moderate forms of asthma are associated with eosinophilic inflammatory cells in the BAL fluid or sputum and TH2 cytokines (IL-4, IL-5, and IL-13) with increased serum IgE (25). Beside TH2 cells, group 2 innate lymphoid cells (ILC2s) play a crucial role in mediating the type 2 immune response through production of IL-5 and IL-13 when activated by alarmins IL-25, IL-33 and thymic stromal lymphopoietin (TSLP) in response to epithelial cells damage induced by viral infections or allergens (Figure 2) (38). Severe forms of asthma have been associated with mixed granulocytes or neutrophilic inflammation accompanied by T helper 17 (TH17) cells and its associated cytokines [IL-17A, IL-6, granulocyte colony stimulating factor (G-CSF), tumour necrosis factor α (TNF-α), and C-X-C motif ligand 8 (CXCL8)] (39). TH2 high endotype has been observed with much higher airway nitric oxide, airway and peripheral blood eosinophils compared to TH2 low endotype (17). TH2 high endotype groups have a high IL-13 transcriptional signature in lung epithelia and periostin in blood (37). These group of patients also tend to be more responsive to corticosteroids, although there are subgroups of a considerable size that have been reported to require high doses of corticosteroids to control asthma (37). More recent studies in adult asthma has shown a non-redundant role for T helper 1 (TH1) cells and its associated cytokine IFN-γ, and chemokines, CXCL10, MIP-1A (40). In this setting TH1 cells were associated with severe forms of asthma, contrary to the long-held notion that these cells counter and protect against developing asthma (Figures 2, 3).

In children, heterogeneity of asthma is less explored partly due to difficulties in obtaining bronchoscopy procedures at that age and limited ability of children to induce sputum. Children are often on high dose inhalable or oral corticosteroids which dramatically change the immune inflammatory profile in the lung (41, 42). In a few studies that have looked at heterogeneity of the asthma inflammatory profile in children, 4 distinct categories could be identified, and these included an eosinophilic type, mixed granulocytic infiltrate, and neutrophilic type (42, 43). Children with the neutrophilic type of asthma were generally found to be younger (between 3 and 9 years old). The neutrophil infiltrate coincided with detection of viral and bacterial pathogens, mainly rhinovirus and *Haemophilus influenza* A, respectively (43). Neutrophil inflammatory phenotype was far more heterogenous and further divided into sub-groups, but generally associated with steroid-refractory type of asthma. Neutrophilia in the BAL fluid of children was associated with increased CXCL8, CXCL10, G-CSF, and IL-17. Interestingly, CXCL8, the highest predictor of neutrophilic inflammation was not associated with presence of viral or bacterial pathogens, suggesting that respiratory infection may not be a main driver of this chemokine response and that infection and cytokines may drive neutrophilic asthma independently (43). In another study, neutrophils were found to be increased in children with severe therapy refractory asthma (STRA), which correlated with increased IL-8 production by primary bronchial epithelial cells from the same children (44, 45). Interestingly, intraepithelial neutrophils but not overall BAL neutrophils were associated with improved lung function (44). IL-17A was found to act indirectly in promoting intraepithelial neutrophil influx, by stimulating increased expression of its receptor IL-17RA in epithelial cells which in turn released more IL-8 to chemo-attract neutrophils. This particular study in children paints a very complex and contradictory picture of TH17 cells in neutrophilic asthma, different to what is observed in adults. T helper 1 cells have been shown to be present in both non-allergic and allergic children with severe asthma (46). These cells were found within the TH2 milieu and dominated in BAL fluid rather than blood, secreting signature cytokines IFN-γ and CCR5 and positively correlated with common allergen-specific IgE secretion. The nature of their development and maintenance in such an environment is unclear, but it is thought that plasmacytoid dendritic cells secreting type I interferons may be the main driver of these cells (Figure 2). Other cell types that have been found to be associated with steroid refractory asthma in children are ILC2s. These cells are sparse, express ST2 and are thought to be the main producers of IL-13. More recent studies showed ILC2 to be associated with steroid resistance in children and are sensitive only to systemic corticosteroids (47–49). CD45RO expression by inflammatory ILC2 has been linked to acquisition of steroid resistance in asthmatics (50).

## Poor Lung Function at Early Age and the Development of Asthma in Children

The period of 0–6 years is crucial, as it is during this phase that the majority of asthma cases are reported (51, 52). Poor lung function at infancy has been identified as one of the predisposing factors of childhood-onset asthma (53, 54). Wheezing in the first year of life is a key feature of impaired lung function resulting from respiratory infections which are recurrent during childhood (Figure 1). In a longitudinal study by Martinez et al. (55) where they followed up children from birth till the age of 6 years, they reported that almost half of the children enrolled in the study had recurrent wheezing episodes by the time they reached age 6. They also presented with signs of diminished lung function (55). They further noted respiratory infection in lower airways by the age of 3 as the leading cause of wheezing which they associated with asthma predisposition. Over two decades later, a follow up study led by Tai et al. (56) further reported wheezing at infancy as a risk factor for subsequent asthma development in adults who were followed up for 50 years (56). Considering that asthma in adolescence is linked to wheezing during the first 6 years of life and subsequent defective lung function, it is therefore within reason to infer that poor lung function in early life does not only predispose children to asthma development, but may be the reason behind disease severity in adults (56, 57).

Several risk factors have been associated with poor lung function in children which leads to poor quality of life. These include maternal smoking, preterm birth, birth weight, heavy use of broad antibiotics at an early age, air pollution PM_10_, respiratory infections and in some parts of the world human immunodeficiency virus (HIV) exposure *in utero* (58). The mechanisms of how these environmental factors influence lung function are unclear. There has been some suggestions that thickening of the reticular basement membrane of the airway smooth muscle in adolescence may be linked to early functional changes that happen in the first few years of life (59). In a study by Altman et al. (60) using RNA sequencing in nasal and blood samples from children with poor lung function, 2 modules were related to asthma exacerbations and these included SMAD-3 network cluster which is related to epithelial differentiation. This network also included genes involved in wound healing and epithelial differentiation such as transforming growth factor beta (TGF-β), KLF4 and FZD1. These studies have been further validated in animal models, where mice lacking TGF-β in epithelial cells fail to induce AHR and ILC2 activation (61, 62). Other modules associated with asthma exacerbations in children included extracellular matrix (ECM), which was mainly dominated by genes such as iNOS, collagen associated genes COL1A1, CAV1, CFTR, and EPHA1 (60).

In this group of children with exacerbations of asthma, they also found an eosinophil activation/mucus hypersecretion module, which was mainly dominated by genes associated with mucus production, MUC5AC, the respiratory proliferator marker KRT8 and genes associated with eosinophil surface marker and activation, CD9 (60). There were also genes associated with eosinophil identity, IKZF2 and also genes associated with epithelial mesenchymal communication in asthma such as FGFR2. Interestingly, some of these genes (IKZF3, PGAP3, GSDMA, GSDMB, ERBB2, and ORMDL3) fall within 17q21 locus which is linked with high risk of children developing early wheezing episodes due to viral and fungal exposure (63). The subsequent study that focused on ORMDL3 proposed that its mechanism is related to the regulation of ICAM1 and epithelial response to stress due to detection of unfolded proteins, which could explain higher vulnerability toward RV infections and an important mediator of AHR induced by fungal allergens (64). Asthma is a heritable disease and host genetics play a crucial part in susceptibility to the disease, for more detailed reviews on this topic we refer the reader to this review article (9).

## Asthma Related Infection

### Association Between Viral Infections and Early Childhood Asthma

The association between viral infections and early childhood asthma is an area of research interest, and advances in virus detection methods such as more sensitive polymerase chain reaction (PCR) have propelled early detection of different viral genotypes (65). Host genetics and other external environmental agents have been associated with asthma exacerbation in young children. Viral infections are thought to be the major triggers of asthma exacerbation, accounting for 80–95% in children compared with 75–80% in adults (66). Respiratory viral infections are important causative agents of many lower tract respiratory illnesses in early childhood ranging from wheezing episodes to more severe bronchiolitis which could lead to later development of asthma (67). Respiratory syncytial virus and rhinovirus are the most common and frequently detected viral exacerbation in newborns (68). Initially, RSV was thought to be a major etiologic agent of viral induced exacerbation, however, subsequent studies have shown that RV dominates after 12 months of life (Figure 1) (13). Thus, this suggests that RSV is the major cause of exacerbation in the first year of life after which RV takes over (9).

### Respiratory Syncytial Virus

Respiratory syncytial virus is a lipid-enveloped negative-stranded RNA virus, which belongs to the Paramyxoviridae family. It is known to cause the majority of respiratory infections in children under the age of 1 year (69). There are two major antigenic groups of RSV, A and B, with about 10 antigenic A genotypes and 13 antigenic B genotypes (70). The severity of RSV infection varies, with most children experiencing relatively mild infection and only 2–3% requiring hospitalisation (71). Children that are particularly at higher risk are those born prematurely, with immunodeficiency, with neuromuscular disorders or those with chronic lung or congenital heart diseases (70, 72). The attachment protein (G) and the fusion protein (F) are the two main surface glycoproteins which play a significant role in the pathogenesis and infectivity of RSV, making them the main targets for the host humoral immune defence (73). Humanised monoclonal antibodies directed against F glycoprotein can decrease the risk of hospitalisation due to RSV infection in pre-term infants (74). These findings formed the basis for potential vaccines or antiviral drugs currently in clinical development.

RSVs bind to CXCR3 on the surface of respiratory mucosal cells through their surface proteins (G and F) which initiates attachment and fusion of viral envelope with the host cytoplasmic membrane leading to internalisation (75) (Figure 2). RSV products such as ssRNA and protein are detected by both nucleic and protein pattern recognition receptors which initiate signals leading to transcriptional factor activation and type 1 and III secretion (76). The ability of RSV to penetrate smaller airways allows it to spread to deeper parts of the lung where they can replicate in type 1 pneumocytes (70, 73). It is the combined effects of hyper immune activation by innate cytokines and viral replication in many parts of the lung that lead to apoptosis of epithelial cells, sloughing and mucus hypersecretion airway obstruction and wheezing (77).

### Rhinovirus

Rhinovirus is a small sized positive non-enveloped strand RNA virus, which belongs to the Picornaviridae family (78). It is broadly classified into three major serotypes according to its capsid binding receptors and genomic sequences, namely: RV-A, RV-B, and more recently discovered RV-C (78). There are over 80 RV-A serotypes, 32 RV-B serotypes and 65 RV-C serotypes. RV-A and RV-C are the most likely cause of severe wheezing and asthma exacerbation in children (79). Improved techniques have allowed for easy detection of several RV serotypes, however, structural and genetic variability across all RV serotypes has hampered efforts to develop effective vaccines or antiviral drugs against them (70). Both drugs aimed at capsid protein or 3C protease inhibitors are not effective against all serotypes (70). Effective future drugs and vaccines are likely to target cross-reactive antigens and possibly multiple antigens.

RV normally causes the common cold in children with up to 35% asymptomatic individuals testing positive for RV (80). RV does not induce chronic infections in healthy individuals, although systemic immune responses have been observed in children presenting with wheezing (80). In adult human challenge studies with RV, asthmatics were found to have increased exacerbations shown by increased type 2 inflammation (Figure 2). Mechanistically RV induced release of host dsDNA and damaging NETosis causes lung tissue damage and airway hyperreactivity (Figure 2) (81). These mechanisms, although not tested in children are likely to be the main contributing factors in early wheezing.

Upon RVs entering the host they bind to mucosal epithelial receptors with RV-A preferentially binding to intracellular adhesion molecule 1 (ICAM-1), RV-B to low density lipoprotein receptor (LDL-R), and RV-C binding to cadherin-related family member-3 (CDHR3) (70). CDHR3 is a risk factor for asthma and its polymorphisms particularly rs6967330 are associated with acute severe exacerbations in children (82). Binding of the viral capsid to these innate receptors such as TLR-4 leads to activation of innate alarmins such as those seen during allergic asthma such as IL-25, IL-33, and thymic stromal lymphopoietin (TSLP) (Figure 2). The release of these cytokines initiate a cascade of inflammatory cells which leads to epithelial permeability, loss of epithelial barrier integrity, release of host DNA and further entry of viral proteins (76, 83). It is the combination of these pro-type 2 environment and enhanced absorption of other allergens and pathogens that lead to impaired lung function, airway obstruction and airway hyperresponsiveness (76, 83).

### Influenza Viruses

Influenza viruses are envelope medium-sized negative sense RNA viruses with eight segmented genomes belonging to the Orthomyxoviridae family (84). There are four groups of influenza viruses: influenza A, B, C, and D. All the four strains have been reported to infect and cause diseases, IAV is the major and common pathogenic strain in humans (85). Morphologically, these viruses are spherical to ovoid-shaped which contain two major surface glycoproteins hemagglutinin (HA) and neuramidase (NA) at 4:1 ratio (86). There are about 16 subtypes of HA (H1–H16) and 9 subtypes of NA (N1–N9) discovered so far, and among all these strains, only H1N1 and H3N2 are known to infect humans and cause seasonal influenza (87). At least 4 IAV pandemics that claimed over 50 million lives have been documented worldwide since the first H1N1 outbreak in 1918, followed by H2N2 in 1957 and H3N2 in 1968 (88). A more recent outbreak happened in 2009 due to H1N1 which killed an estimated 280,000 (157,700–575,400) people worldwide according to Centre for Disease Control.

Influenza viruses remain one of the leading causes of severe pneumonia or bronchitis early childhood, despite universal availability of influenza vaccines that prevent infections in under 5 years (89). Direct evidence both experimentally and epidemiologically have failed to show clear associations between influenza viral infection and asthma exacerbation in early childhood due to the inconsistencies and contradictory results in animal models or laboratory experiments (90).

Influenza A virus enters the lung and binds to sialic acid on the surface of alveolar epithelial cells through its HA component, which triggers the endocytosis of the virion into the host cell (Figure 2) (91). After viral replication in the nucleus, viral assembling, budding and scission take place at lipid raft in the plasma membrane (92). The newly formed virion binds sialic acid receptors, and later cleaved by NA component of IAV to release the viral progeny that can infect other cells (93). The viral replication that hijack eukaryotic machinery induces both innate and adaptive immune responses, which triggers the release of several immune cytokines including IFNs, TGF-β, IL-10, and IL-13, to mount an anti-viral response that is beneficial to the host (88). However, the prolonged presence of these cytokines in the lungs, especially IL-13, have been associated with asthma exacerbation (94).

### Coronavirus

Coronavirus disease 19 (COVID-19) is caused by severe acute respiratory syndrome coronavirus-2 (SARS-CoV-2), which belongs to the family of *Coronaviridae* and order of *Nidovirales*. SARS-CoV-2 is the latest discovered member of the coronavirus family which preferentially affects respiratory tract (nose and lungs), leading to common cold, bronchitis, and pneumonia (95). Since the first reported paediatric case of a 10 year old boy in Wuhan, a multitude of coronavirus cases have been reported globally in young children (96).

Initially, there was a growing concern of childhood asthma being a risk factor for severe COVID-19 outcomes. Subsequent studies, however, have not supported this view arguing that paediatric asthma could rather protect against COVID-19 (97). This is due to the presence of TH2 cytokines and frequent use of inhaled corticosteroids (ICSs), which suppress angiotensin converting enzyme 2 (ACE-2) (98, 99). SARS-CoV-2 enters the human host through the nose and travels to alveoli in the lower respiratory tract, where it attaches to ACE-2 receptors on type I and II alveolar cells through proteolytic activity of transmembrane protease serine 2 (TMPRSS2) (Figure 2) (100, 101). SARS-CoV-2 once bound to ACE-2 receptor triggers a cascade of proinflammatory cytokines such as IL-6 which have been associated with exuberant immune responses leading to cytokine storm and severe disease outcomes in the elderly (102).

Childhood asthma has been shown not to be a risk factor for COVID-19 disease complications. These could be attributable to (1) cross-immunity from other similar coronaviruses; (2) lower risk of non-communicable diseases such as cardiovascular diseases, (3) low expression of ACE-2 receptors and TMPRSS2 in both nasal and airway epithelial cells (100): and (4) and lastly, type 2 cytokines such as IL-13 and use of corticosteroids which are thought to suppress ACE-2 receptor expression *via* downregulation of type I interferon (Figure 2) (103, 104).

### Human Immunodeficiency Virus

Despite significant progress made in reducing mother-to-child human immunodeficiency virus (HIV) transmission in Sub-Saharan Africa, where almost 30% of pregnant women are living with HIV (105), there remains a huge burden of HIV-exposed uninfected (HEU) infants. These HEU children are in great danger of developing respiratory infections, as well as a high chance of morbidity and mortality due to invasive pneumonia and respiratory syncytial virus (RSV) in early life (106–109). There are several risk factors associated with prevalence and mortality in HEU children including, pre-term birth, low birth weight, low maternal CD4 count, bacterial meningitis and severe pneumonia (110, 111). Perinatal exposure to anti-retroviral therapy (ART) has been associated with abnormal mitochondrial morphology and subclinical defects in hematologic parameters in HEU children, which may persist throughout childhood (112, 113). In addition, it has been hypothesised that poor lung growth and functions *via* dysregulation of metabolic pathways in HEU are linked to maternal antiretroviral therapy (114). However, initiation of ART prior to pregnancy has shown to improve maternal health, as well as reduce the risk of respiratory infections, morbidity and hospitalisation in these children in the developed countries (115). What seems to be the main contributing factor in HEU is immune defects in both humoral and cellular responses which may be the main contributor to respiratory infections and poor lung function. Compared to HIV unexposed uninfected children, HEU demonstrated lower antibodies to several pathogens, which could potentially increase early life susceptibility to infectious agents (116). HEU infants also show increased lymphocyte phenotypes which leads to deficiency in the production of TH1 cytokine, IFN-γ, which limits activation of antigen presenting cells (APCs) (117, 118). This deficiency in TH1 cells has been shown to lead to pneumonia caused by *Pneumocystis jirovecii* in the first 6 months of life in at least a 3rd of HEU infants (118).

Whether HEU is a risk factor for asthma is currently unclear, but what seems to be common is poor lung function and susceptibility to infections in the first 6 months. One example is RSV infection which resulted in prolonged hospitalisation and deaths in HEU children (107). RSV is also the major inducer of asthma exacerbation in young children, future studies should investigate the link if any, between HEU and asthma exacerbation in the first few months of life.

## Microbial Components and Asthma

The bacterial cell wall is composed of various components, each with a contributory role in protecting and keeping the bacteria's structural integrity. Gramme-negative bacteria bear, among other molecules, endotoxins, which are crucial to the cell's survival and function (119). Endotoxins are lipopolysaccharides (LPS) ubiquitous in both the outdoor and indoor environment, with the main source of these molecules being livestock, pets, etc. (119, 120). Despite their toxic propensity, the beneficial influence that these molecules have as therapeutic intervention for various conditions has been widely demonstrated (120, 121). In asthma, specifically, a beneficial role has been noted in several studies where endotoxins derived from farm animals have been associated with a decrease in asthma development in children (122–125). In Germany, one of the large studies that enrolled 10,163 children of school going age (5–7 years) observed an inverse relationship between the development of allergic diseases, including asthma and animal exposure (123). In this study they showed that the repression of atopic disease manifestation was a consequence of bacterial-derived molecules attributable to consistent and augmented animal exposure, which is inherent for children who grow up in farms. This substantiates the hygiene hypothesis which states that increased infections or dirty environments in early-life lead to reduced autoimmune and allergic disorders (126). The hygiene hypothesis is, however, a controversial subject, with various studies arguing for and against it (127–131).

Despite the apparent instrumental role of endotoxins against atopy, as evidenced by the above-mentioned studies, literature has also established the negative effect these molecules possess. Endotoxins are important immune response inducers, as such, they have been implicated in the development of allergic diseases including asthma (119, 132, 133). Upon inhalation endotoxins are recognised by PRRs such as Toll-like receptor 4 (TLR-4). The lipid A portion of endotoxins drives the ensuing immune response by triggering a proinflammatory response characterised by cytokine production and cellular responses (134, 135). In 1989 a study evaluating lung function following endotoxin exposure revealed a decrease in carbon monoxide diffusion, increased bronchial reactivity and a slight decrease in FEV_1_ (136), which are all key features of asthma. However, it has been suggested that responses observed following endotoxin exposure are dose dependent, with a low dose conferring protection to infections and a high dose capable of eliciting a detrimental response such as septic shock (Figure 3) (136–138). It is therefore clear that the interaction between genetics and environmental factors plays an influential role on the outcomes of allergic responses.

Another ubiquitous bacterial component is flagellin which is a protein, not unique to, but mostly present in Gramme-negative bacteria. Besides its incorporation as an adjuvant in vaccine design, this protein has proinflammatory abilities as it is recognised by the innate receptor TLR5, expressed on the surface of innate immune cells (139). In comparison to LPS, another potent immune inducer, it has been found to be superior as a mucosal immune cell activator (140–142). Similar to LPS, studies have shown conflicting evidence on the role of flagellin in allergic diseases including asthma (Figure 3). While some authors have raised the importance of this protein in the suppression of allergic response by supporting a more regulatory response, others have associated this very same molecule with inflammatory responses that trigger asthma. For example, in an asthma mouse model it has been demonstrated that a higher dose of flagellin exposure confers protection against asthma development through the induction of immunoregulatory T cells and dendritic cells (rDCs) *via* the TLR5 pathway (143). This inhibitory effect of flagellin on asthma was accompanied by reduced AHR and airway inflammation. The effect of flagellin or its receptor, TLR5, on early-onset asthma remains elusive in humans, specifically in children, with only one study on atopic dermatitis during infancy correlating reduced risk of atopic dermatitis development to increased expression of TLR5 in cord blood (144). Considering that TLR5 level of expression inversely correlates to atopic dermatitis, and developing atopic dermatitis at infancy partially predisposes individuals to childhood asthma (145), it is therefore fitting to propose that TLR5 stimulation at early life stages serves a protective role to asthma development (Figure 1). However, a contradictory role of flagellin was first reported by Wilson and colleagues in 2012 who, using a mouse model found flagellin exposure detrimental as it drove a TH2 biassed response which in turn promoted asthma manifestation in mice (146). Furthermore, they detected high levels of flagellin-specific antibodies in asthmatic patients suggesting that exposure to flagellin could be a risk factor for asthma.

It is clear that the role of microbial components and their associated receptors in asthma development is contradictory and strongly influenced by the experimental setting including dose and timing. A very important question remains, what does this mean for humans? The concentration of allergens to which humans are exposed to cannot be controlled nor can the age at which this exposure occurs.

## Bacterial Infection and Asthma

Bacterial respiratory infection at early childhood is closely associated with asthma development. While most studies have referenced viruses such as rhinovirus and respiratory syncytial virus as important inducers of asthma in early life, we now know that bacterial colonisation also drives disease exacerbation (147). Among several bacterial species that have been implicated in asthma development, *Haemophilus influenza* and *Streptococcus pneumoniae* are the most cited (148, 149).

### Streptococcus pneumoniae

Pneumococcal infections are key contributors to the high mortality rate among children in low- and low-and-middle-income countries (150, 151). *Streptococcus pneumoniae*, the cause of pneumococcal infections, is a Gramme-positive bacterium encapsulated by polysaccharides (152). The bacteria normally colonise the upper respiratory tracts and exist as a commensal organism in healthy hosts. However, it can cause disease in children under the age of 2 years and in adults over the age of 65 years and this is mainly due to the immaturity of the immune system and defectiveness that comes with age (153). *S. pneumoniae* can cause mild to more severe disease from local empyema, necrotising pneumonia to lung abscesses or systemic complications such as sepsis, septic shock, metastatic infection, multiorgan failure, acute respiratory distress syndrome, intravascular coagulation, and rarely death in children (154). *S. pneumoniae* infection can be treated with antibiotics which prevent prolonged hospitalisation and necrotising pneumonia in children (155). The introduction of pneumococcal conjugate vaccine PV7 and more recently PV13 which targets serotypes 1, 3, 5, 6A, 7F, and 19A has significantly reduced incidence and rates of hospitalisation due to empyema and necrotising pneumonia (156). Several studies have associated *S. pneumoniae* colonisation with allergic airway disease. In Denmark, a prospective study enrolled a total of 411 children born of asthmatic mothers that were followed from 4 weeks till the age of 3 years (157). Upon investigation it was discovered that wheezy episodes in the first 3 years of life were significantly associated with bacterial infection and *S. pneumoniae* was among the five bacterial species that were identified (Figure 3). Not only does colonisation with *S. pneumoniae* predispose children to wheezing but there are also implications for asthma development propelled by bacterial infection (158). Corroborating these findings is a neonatal mouse model, where an increase in airway hyperreactivity and an influx of inflammatory cells following *S. pneumoniae* induced pneumonia (149). These studies serve as proof of the ability of bacterial infections to cause or exacerbate airway allergic disease in neonates.

A different stance on the role of *S. pneumoniae* has been raised by several studies, whereby instead of a harmful effect, it can be used for therapeutic purposes (159, 160). In fact, this is not a new discovery, back in the 1980s it was noted that when asthmatic children were vaccinated with pneumococcal vaccine, asthma attack incidences were reduced by 56% (161). The action by which *S. pneumoniae* confers protection against asthma development and progression is through the suppression of both TH1 and TH2 responses, reduction of airway inflammatory cell recruitment and abrogated airway obstruction (160). Furthermore, an inverse correlation between the intensity of symptoms experienced by individuals during an asthma attack and *S. pneumoniae* colonisation has been reported. Although mechanistic studies still need to be carried out to fully elucidate this phenomenon, TNF-α is implicated as the immunoregulator of *S. pneumoniae* induced protective effects (159).

### Haemophilus influenzae

*Haemophilus influenzae* is a gramme-negative anaerobic bacterium composed of both capsulated and non-capsulated strains responsible for a range of illnesses, from mild ear infection to invasive diseases like sepsis (162, 163). The classification of this bacterial species is based on the presence or lack of a polysaccharide capsule, with only one group of encapsulated type referred to as non-typeable *H. influenzae* implicated in various inflammatory disease exacerbation (163, 164). Naturally, *H. influenzae* forms part of healthy individuals' upper respiratory tract microbiome, with colonies detected within the first year of life, progressively expanding through to adulthood (165). Similar to what happens when *S. pneumoniae* predominates, a microflora that is dominantly characterised by *H. influenzae* is seen in individuals with airway diseases including asthma (166). Furthermore, *H. influenzae* has the ability to attach on several receptors of the airway epithelium, thus facilitating augmented inflammatory responses. The Intercellular adhesion molecule 1 (ICAM-1) is one of the receptors, whereby the interaction between this receptor and *H. influenzae* is fostered by the major outer membrane protein P5 fimbria (Figure 3) (164). The pathogenic role of *H. influenzae* extends beyond its involvement in the exacerbation of inflammatory conditions such as chronic obstructive pulmonary disease (COPD) and sinusitis (163). *H. influenzae* colonisation is also responsible for a substantial number of cases of invasive diseases in children below the age of 5 years and in adults over 50 years. Though the introduction of *H. influenzae* type b vaccine has had a great impact over the past 2 decades in reducing incidence rate of *H. influenzae*-related diseases, *H. influenzae* still possesses a great challenge as is it implicated in both asthma inception and disease exacerbation.

*H. influenzae* is among the top 5 mostly frequently detected pathogenic bacteria in children during early-life stages and is a common feature of neutrophilic asthma (158, 167). Experimentally, *H. influenzae* promotes a predominant neutrophilic inflammation through IL-17 induction, which in turn supresses TH2 responses, consequently propelling the development of non-controllable asthma (168). Moreover, exposure to *H. influenzae* over a long period of time has been proven detrimental as it triggers a TH17-dependent neutrophilia and the subsequent defective regulatory T cells (Tregs) response, leading to chronic inflammation and airway remodelling (Figure 3) (169). In humans, though the population of neutrophil-driven asthmatics is disproportionately comprised of adults, *H. influenzae* colonisation is one of the features of childhood neutrophilic asthma, the disease form that is usually unresponsive to treatment (158).

## Microbiome and Asthma

The microbiome is a significant part of our defence system made up of a community of commensal microorganisms whose main responsibility is to maintain immune homeostasis (170). However, an occasional imbalance in the microbial community occurs in certain individuals, and when this happens, a superfluous immune response is triggered leading to several immune disorders. Studies have associated particular imbalances with asthma development. For example, a shift from a predominantly Firmicutes and Proteobacteria to a Bacteroidetes dominant microbial community in neonatal mice lungs induces Tregs (171). The importance of T cell differentiation into Tregs and their role in regulating allergic responses is well-documented (172–174). Additionally, lung microbiota formation is one of the identifiable factors that set the trajectory of our respiratory health (Figure 3). Among the first bacterial species to appear (at 24 h post-birth) are communities of *Staphylococcus* spp., *Streptococcus* spp., and *Neisseria* spp., to name a few (175). Within the first 2 months of life the microbiome gradually diversifies and matures to a profile comparable to that of adults. Immediately following the peak of diversity and maturity, population readjustment occurs, with *Staphylococcus* spp. presence showing a decline as early as 7 weeks postpartum (175), which is replaced by an abundance in *Streptococcus* spp., among other bacterial species. *Streptococcus* spp. is one of the species well-known for its association with infant-wheezing at 1 month (158), a known predisposing factor of asthma development (Figure 3). The presence, as well as the absence of these colonising bacterial species has been directly correlated to the control or lack thereof of allergic responses. For example, the aforementioned shift to a more Bacteroidetes biassed population triggers allergen tolerance through Treg induction in a programmed death ligand 1 (PD-L1)-dependent manner (171).

The development of early microbiota is dependent on several factors. While neonatal microbial profile is vastly influenced by the mode of delivery at birth, other studies have also shown how the use of antibiotics can lead to an antibiotic-driven microbiome dysbiosis which is associated with an increase in asthma burden in children (176). A prospective study looking at the impact of mode of delivery on microbiome development found that delivery by Caesarean section affects the rate at which the microbial communities are formed as well as the abundance of respiratory-health related microbiota (177). The consequence of the aforementioned events is said to influence respiratory health from childhood through to adulthood, which then corroborate the contributory role that lung dysbiosis has on asthma inception. Antibiotics, on the other hand, do not only disturb lung homeostasis by acting directly on commensal bacteria, they also compromise the ability of the immune system to distinguish between self and non-self-antigen (178). Since commensal bacteria are essential triggers of the immune system's regulatory cells, when antibiotics are administered, they can, non-selectively, strip away the essential bacteria, leaving the host susceptible to allergic disease development. Several studies have correlated asthma development to early-life antibiotic exposure (178–181). This then evidently demonstrates the importance of bacterial colonisation at infancy and the role it plays as a determinant of respiratory health and potential asthma development.

Mechanisms of how microbiota can limit asthma development at an early age are not fully understood. Some studies have suggested that microbial metabolites released by good microbiota act directly on pathogenic bacterial species to limit their growth by either reducing essential nutrient availability or promoting toxic by-products that can inhibit microbial growth (182, 183). Mechanistically, gut microbiota fuelled by selective antibody pressure enhance capacity of L-tyrosine metabolising species, which in turn increases levels of p-cresol sulphate. P-cresol sulphate was able to reduce allergic asthma through reduction of epithelial derived CCL20, a chemokine known to drive type 2 allergic airway inflammation.

## Asthma Mouse Models

Animal models have proven to be critical in asthma research, simple because they develop features of the disease similar to humans when exposed to ovalbumin (OVA) complexed to adjuvant alum or house dust mite, a more complex and relevant allergen (184–187). Key pathophysiological features of asthma that can be reproduced in animals include increased allergen-specific IgE, increased eosinophils, TH2 cells and airway hyperreactivity which can be induced by bronchoconstrictors (187). Availability of a wide array of genetically deficient and transgenic mice strains is attractive for studies of pre-clinical disease mechanisms. Although adult mouse studies have been used successfully for over 5 decades to mimic asthma in humans, models that mimic early development, a crucial stage in disease onset has been difficult (188). To fully appreciate early determinants of asthma pathophysiology, several studies have recapitulated these early events in animal models of allergic asthma (188, 189). Saglani et al. (188) developed a neonatal mouse model that has been widely adapted. In this study, they used a more natural route where 3-day old mice were sensitised intranasally with HDM to investigate early-onset of asthma (188). At steady state, innate lymphoid cells (ILC2s) producing IL-13 dominate the first 10 days of life in an ST2-dependent manner (190), whereas during HDM exposure CD4 T cells producing IL-13 are thought to be the main contributor toward allergic airway inflammation (41). Interestingly, neonatal 3-day-old mice deficient of ST2 or lacking IL-13 production specifically in ILC2 develop AHR, whereas those lacking IL-13-specifically in CD3 T cells are unable to develop AHR (41). Other studies using *Alternaria alternata*, a fungi mainly associated with steroid resistant asthma in children and adults have shown that this IL-33-dependent allergic airway inflammatory response can only be treated with steroids (190–192). Interestingly, this TH2-biassed immune phenotype is also characterised by M2 macrophages that favour resolution of inflammation and protect the vulnerable developing lung (24). These macrophages are dependent on TGF-β signalling which sustain these cells in the first few days of life and promote TH2 responses (193).

T helper 2 environment dominates early stages of life, partly due to an evolutionary protective response in pregnant mothers which is transferred to offspring mainly to protect against foreign pathogens in a newborn (194). What has been unclear is how children are the most vulnerable to developing allergic responses, even in the presence of constant microbial exposure which is thought to be protective through hygiene hypothesis (126). A recent study in an HDM model elucidated mechanisms that drive early-onset of asthma in neonatal mice. Here the authors showed that when mice are sensitised using HDM and low dose LPS, they respond differently depending on the age (195). While low dose LPS was able to suppress TH2 allergic airway inflammation in adult mice, in neonatal mice the TH2 allergic airway inflammation was sustained (195). Mechanistically, antigen presenting cells in neonates were hypo responsive to LPS through reduced expression of TLR-4. This reduced TLR-4 signal led to reduced TNF-α-dependent activation of migratory DCs, which induced less T-bet transcription and less IL-12. This reduced IL-12 expression led to reduced polarisation of T cells toward TH1 lineage and increased transcription of GATA-3 and TH2 cytokines in neonates (195). This study is well within the premises of the hygiene hypothesis, where the intensity of germ exposure is correlated to protection from allergic disease development.

## Concluding Remarks

Childhood asthma is a chronic disease affecting close to 9 million children in the US alone. The development of asthma is complex and is likely to be influenced by genetics and environment factors *in utero* and postnatally. The lung completes its final maturation in the first 3 years of life which makes it vulnerable to viral and bacterial infections, which can set a detrimental trajectory in lung function. Understanding which factors are critical in poor lung function leading to early wheezing is an important question. The heterogeneity in immune profiles observed in children with asthma has not been explored in detailed, partly due to difficulties in accessing bronchoscopy in children. Non-invasive methods such as bronchial brushing or nasal brushings have proven to be reliable, showing great similarities in gene expression between lung bronchial biopsies and nasal brushings (17, 196). Nasal brushing transcriptomics showing TH2 high and TH2 low asthma, highly correlate with eosinophil levels observed in blood, which suggests that more complex cases of asthma can be detected this way. There is a need to closely monitor birth cohort studies and sample children non-invasively at different time points to fully understand what immunological markers or defects can be correlated with early development of poor lung function and early wheezing. There is also a need to consider other chronic diseases in mothers, such as HIV, which could have negative impact on normal development of children, leading to poor lung function and early wheezing. Animal models are vital in understanding the early development of asthma, and further work in this area to better model human disease may lead to improved therapeutics.

## Author Contributions

SH and FB conceived the idea. NM and PI contributed equally to the writing of this manuscript. NM, PI, FB, and SH wrote the paper. All authors contributed to the article and approved the submitted version.

## Conflict of Interest

The authors declare that the research was conducted in the absence of any commercial or financial relationships that could be construed as a potential conflict of interest.
